# Suppression of Natural Killer cell NKG2D and CD226 anti-tumour cascades by platelet cloaked cancer cells: Implications for the metastatic cascade

**DOI:** 10.1371/journal.pone.0211538

**Published:** 2019-03-25

**Authors:** Christopher D. Cluxton, Cathy Spillane, Sharon A. O'Toole, Orla Sheils, Clair M. Gardiner, John J. O'Leary

**Affiliations:** 1 Department of Histopathology, Trinity College Dublin, Dublin, Ireland; 2 Emer Casey Research Laboratory, Coombe Women and Infants University Hospital, Dublin, Ireland; 3 Trinity Biomedical Sciences Institute, Trinity College Dublin, Dublin, Ireland; 4 The Trinity Translational Medicine Institute, Trinity College Dublin, Dublin, Ireland; 5 Department of Obstetrics and Gynaecology, Trinity College Dublin, Dublin, Ireland; Duke University School of Medicine, UNITED STATES

## Abstract

Tumour cell immune evasion is a principal hallmark of successful metastasis. Tumour cells in the vasculature adopt a platelet cloak that efficiently suppresses the innate immune system by directly inhibiting Natural Killer (NK) cells, which normally function to neutralise spreading cancers. Here we describe two novel mechanisms of tumour cell evasion of NK cell anti-tumour functions. The first, an ‘immune decoy’ mechanism in which platelets induce the release of soluble NKG2D ligands from the tumour cell to mask detection and actively suppress NK cell degranulation and inflammatory cytokine (IFNγ) production, concomitantly. This represents a double-hit to immune clearance of malignant cells during metastasis. The second mechanism, a platelet-derived TGFβ-mediated suppression of the CD226/CD96-CD112/CD155 axis, is a novel pathway with poorly understood anti-cancer functions. We have demonstrated that platelets robustly suppress surface expression of CD226 and CD96 on the NK cell surface and their associated ligands on the tumour cell to further enhance NK cell suppression. These highly evolved mechanisms promote successful tumour immune evasion during metastasis and provide a unique opportunity for studying the complexity of cellular interactions in the metastatic cascade and thus novel targets for cancer immunotherapy.

## Introduction

Cancer is a leading cause of death in the developed world, second only to cardiovascular disease [1]. Greater than 90% of all cancer-associated deaths are caused by metastasis [1], and by extension, metastasised cancer is effectively an incurable disease. Primary tumour cells that intravasate into the peripheral circulation are called circulating tumour cells (CTCs). CTCs represent a promising target for anti-cancer screening and therapy. However, to efficiently detect and target CTCs, a greater understanding of their biology, particularly as it relates to their evasion of the immune system is essential. As such, our study attempts to understand the biology of CTCs by examining how platelets function to promote their evasion of the immune system using *in vitro* models of established tumour cell lines and Natural Killer cells.

In the circulation, cancer cells must overcome physiological barriers. Cancer cells are subject to mechanical shear stress, immunological surveillance by immune cells in the peripheral circulation and cellular checkpoints for apoptosis and senescence. One mechanism by which they overcome these challenges is the adoption of a cloak of platelets onto their cellular surfaces [2]. Cancer cells bind to platelets by GPIIb-IIIa-fibrinogen binding and up-regulate P-selectin on activated platelets [3]. This induces a cascade of platelet activation, resulting in cancer cell-platelet clusters that protect the cancer cells from mechanical and biological clearance [4]. The complex molecular processes of protection are, however, not fully understood. It is believed that this protection is, in part, mediated by the physical barrier of the platelet cloak as many anti-tumour actions require direct contact between the immune cell and the cancer cell target [5]. However, recent data indicates that activated platelets can transfer their major histocompatibility complex (MHC) class I to cancer cells by trogocytosis, which allows the cancer cell to escape immune detection via the newly acquired ‘self’ signal [6]. Moreover, cancer cells activate platelets, inducing degranulation and release of a plethora of immune modulators including potent immune suppressant cytokines, such as transforming growth factor β (TGFβ) [7].

Natural killer (NK) cells are immune cells that function to lyse tumour cells without prior sensitization [8]. They play an important role in the immunosurveillance of tumours by recognizing and eliminating malignant cells, thereby preventing both local tumour progression and metastatic spread [8]. NK cell reactivity is guided by the principles of “missing-self” and “induced-self,” which asserts that cells with low or absent expression of MHC class I (missing-self) and/or stress-induced expression of ligands for activating NK receptors (induced-self) are preferentially recognized and eliminated [9]. These functions of NK cells are complex and are regulated by a range of activating and inhibitory receptors expressed on the NK cell surface [9] and it is a balance of activating and inhibitory signals mediated by these receptors that determines whether NK cell responses will proceed. In the context of cancer cell surveillance and clearance, natural-killer group 2, member D (NKG2D), CD226 (DNAM-1) and CD96 (TACTILE) bind to ligands of cellular stress often overexpressed on malignantly transformed cells [9]. In response, NK cells degranulate and produce pro-inflammatory cytokines to stimulate an inclusive immune response to the transiting tumour cells [10].

In this study, we report that platelet cloaking of tumour cells promotes the loss of the NKG2D ligands, MICA and MICB, from the surface of tumour cells, inducing their secretion into the tumour microenvironment where they suppress NKG2D receptor expression on NK cells. This represents a sophisticated two-hit mechanism for platelet-mediated tumour cell evasion from immune defences and reveals an important therapeutic target to disrupt cancer cell functions during metastasis. Additionally, we describe the novel mechanism of platelets and their soluble molecules downregulating the receptors and ligands of the CD226/CD96-CD155/CD112 axis, further inhibiting NK cell functions and promoting tumour cell survival. This represents a potent mechanism of immune-evasion in cancer. We therefore suggest that better understanding of the molecular basis of NK cell immune evasion by tumour cells in circulation will enable the development of future therapeutics targeting the metastatic cascade in patients with late-stage disease.

## Materials and methods

### Ethics statement

Recruitment of participant blood donors for this study was approved by the School of Biochemistry & Immunology, Level 1 REC in Trinity College Dublin and written informed consent was obtained from all donors prior to phlebotomy. Additionally, all experiments were performed in accordance with relevant guidelines and regulations.

### Reagents

Anti-CD3-PerCP, CD56-PE, CD107a-FITC and CD42B-APC, including their IgG controls, were obtained from BD Pharmingen (San Diego, CA). Anti-IFNgamma-PE-Cy7, NKG2D-APC were obtained from eBioscience (San Diego, CA). Anti-MICA (AMO1) and MICB (BMO2) were obtained from BAMOMAB (Germany). Anti-mouse alexa-fluor-488 was obtained from Life Technologies (California, USA). Anti-CD226-FITC, Anti-CD96-PE, anti-CD155-FITC and anti-CD112-APC were obtained from Biolegend (California, USA). A TGF-β-1 neutralising antibody and recombinant TGF-β-1 was obtained from R&D systems (Minnesota, USA). Recombinant MICA and MICB were obtained from R&D systems (Minnesota, USA). Recombinant Human IgG1 Fc Protein, CF from R&D systems (Minnesota, USA). Thrombin receptor-activated peptide (TRAP) was obtained from Sigma-Aldrich (USA).

### Cell lines

Ovarian 59M and SKOV3 cells and melanoma SK-Mel-28 cells were used as a model system and cultured as previously described [11, 12]. K562 control cells were established and cultured as previously described [13].

### Preparation of peripheral blood mononuclear cells

For each experiment, Peripheral blood mononuclear cells (PBMCs) from healthy donors were used. Each experimental replicate is a representative of different donor. PBMCs were isolated from peripheral blood. Briefly, whole blood was layered onto LymphoPrep (Axis-Shield) and centrifuged at 600 x g for 30 minutes. PBMCs, which formed a white cell layer, were taken and washed with PBS. Red cells were eliminated by incubating the cells in red blood cell lysis buffer (Life Technologies) for 5 minutes at room temperature and washed again in PBS. Washed PBMCs were counted and plated according to each assay’s specifications.

### Preparation of washed platelets

Platelets were donated by healthy donors. Each experimental replicate is representative of a different healthy donor. Blood was collected from donors by venepuncture through a 19-gauge butterfly needle without a tourniquet, to avoid platelet activation. Platelets were prepared as previously described [11].

### Platelet cloaking

The platelet adhesion assay was performed as previously described [11]. Briefly, washed platelets were co-incubated with tumour cells at a ratio of 1000:1 in RPMI media at room temperature under gentle rocking for 1 hour to obtain platelet cloaked tumour cells. To eliminate soluble factors, cloaked tumour cells were washed (3x) with PBS and co-incubated with PMBCs. To obtain soluble platelet cloaked tumour cell releasate, cloaked tumour cells were centrifuged at 1000xg for 5 minutes and the supernatants were used with PBMCs for functional analysis.

### Staining of cell surface molecules for flow cytometric analysis

NK cells were incubated with optimal concentrations of the following anti-human Abs: CD56-PE, CD3-PerCP, NKG2D-APC, CD226-FITC, CD96-PE or the corresponding isotype control Abs (all from BD Pharmingen) in 100 μl of 1% FCS/PBS at 4°C for 20 min.

Tumour cells, with and without platelets, were incubated with anti-human MICA, MICB unconjugated antibodies and alexa-fluor-488 secondary antibody. Staining of tumour cells using primary and secondary antibodies was performed for 1 hour at 4°C. Tumour cells were also stained with CD112-APC and CD155-FITC antibodies and isotype controls in 100 μl of 1% FCS/PBS at 4°C for 20 min.

Cells were washed twice with PBS and acquired on a Cyan flow cytometer (Beckman Coulter, Brea, CA, USA). Events were stored and analysed on the FlowJo software (TreeStar). NK cells were gated from PBMC populations as distinct CD3-CD56+ cells (S1 Fig).

### CD107a staining

A total of 1 × 10^6^ PBMCs were stimulated with 500U hrIL-2 for 18 h in 96-round-bottom well plates. Freshly re-suspended tumour cells (2 x 10^6^) and anti-CD107a-FITC (2 μl/well) or IgG1-FITC (2 μl/well), as a control, were then added. Plates were incubated for 1 h, before the addition of GolgiStop (BD Pharmingen). Plates were incubated for further 3 h before extracellular staining and flow cytometric analysis. All incubations were performed at 37°C.

### IFN-γ intracellular staining for flow cytometric analysis

Cells were stimulated with 500U hrIL-2 for 18 h, the last 4 h in the presence of Golgi-Plug (BD Pharmingen). Cells were washed once in PBS and FcRs were blocked by incubating with 10% human AB serum in 1% FCS/PBS. Cell surface staining was performed as above, followed by intracellular staining with IFN-γ-PE-Cy7 or IgG2a/b-PE-Cy7 (both from BD Pharmingen) as control using the Cytofix/Cytoperm Plus kit (BD Pharmingen) according to the manufacturer’s instructions.

### Functional assays

NK cell activation assays were performed with PBMCs at a PBMC to target cell ratio of 5:1. PBMCs yielded 10% NK cells on average. Functional studies were performed in the presence and absence of blocking antibodies to NKG2D, CD226 or CD96 on NK cells and MICA, MICB, CD155, CD112 tumour cell surfaces respectively. Here, cells were incubated in 1ug/mL solutions of either anti-NKG2D (R&D clone# 149810), anti-MICA (R&D clone# 159227), anti-MICB (R&D clone# 236511), anti-CD226 (BioLegend clone# 11A8), anti-CD96 (BioLegend clone# 92.39), anti-CD155 (BioLegend clone# SKII.4) and anti-CD112 (BioLegend clone# TX.31) for 1 hour at room temperature and washed to remove excess soluble antibody. Recombinant MICA and MICB were used at a final concentration of 10μg/mL for 30 minutes with PBMCs. IgG controls were used for each experiment.

Recombinant TGFbeta was used to pre-treat PBMCs for 1 hour at room temperature prior to functional assay in relevant experiments.

Activated platelets were obtained by incubating washed platelets with 125μg/mL thrombin receptor-activated peptide (TRAP) for 30 minutes at room temperature under gentle rocking conditions. Activated platelets, and their releasate, were then used in functional assays as indicated within the text.

### ELISA

Detection of soluble MICA and MICB was performed using DuoSet ELISA development system from R&D Systems, according to the manufacturer’s instructions. All concentrations are expressed as mean ± SEM of triplicates.

### Real time PCR

Cancer cells were incubated for 24 hr in standard cell culture conditions with media alone or with media containing washed platelets, with a final cancer cell-platelet ratio of 1:1000. Total RNA was extracted from the samples using the miRVana Kit (Life Technologies, Foster City, CA, USA), according to manufacturer’s protocol. ADAM10, ADAM17, ADAM19 and GAPDH mRNA expression levels were evaluated by TaqMan RT-PCR. The data was analysed using the comparative Ct method; where ADAM10, ADAM17 and ADAM19 mRNA expression was normalised to that of GAPDH and calibrated to that of untreated cells to establish the relative level of mRNA expression.

## Results

### Platelet cloaking facilitates evasion of ovarian and melanoma cancer cells from NK cell anti-tumour activity

**Fig 1:**
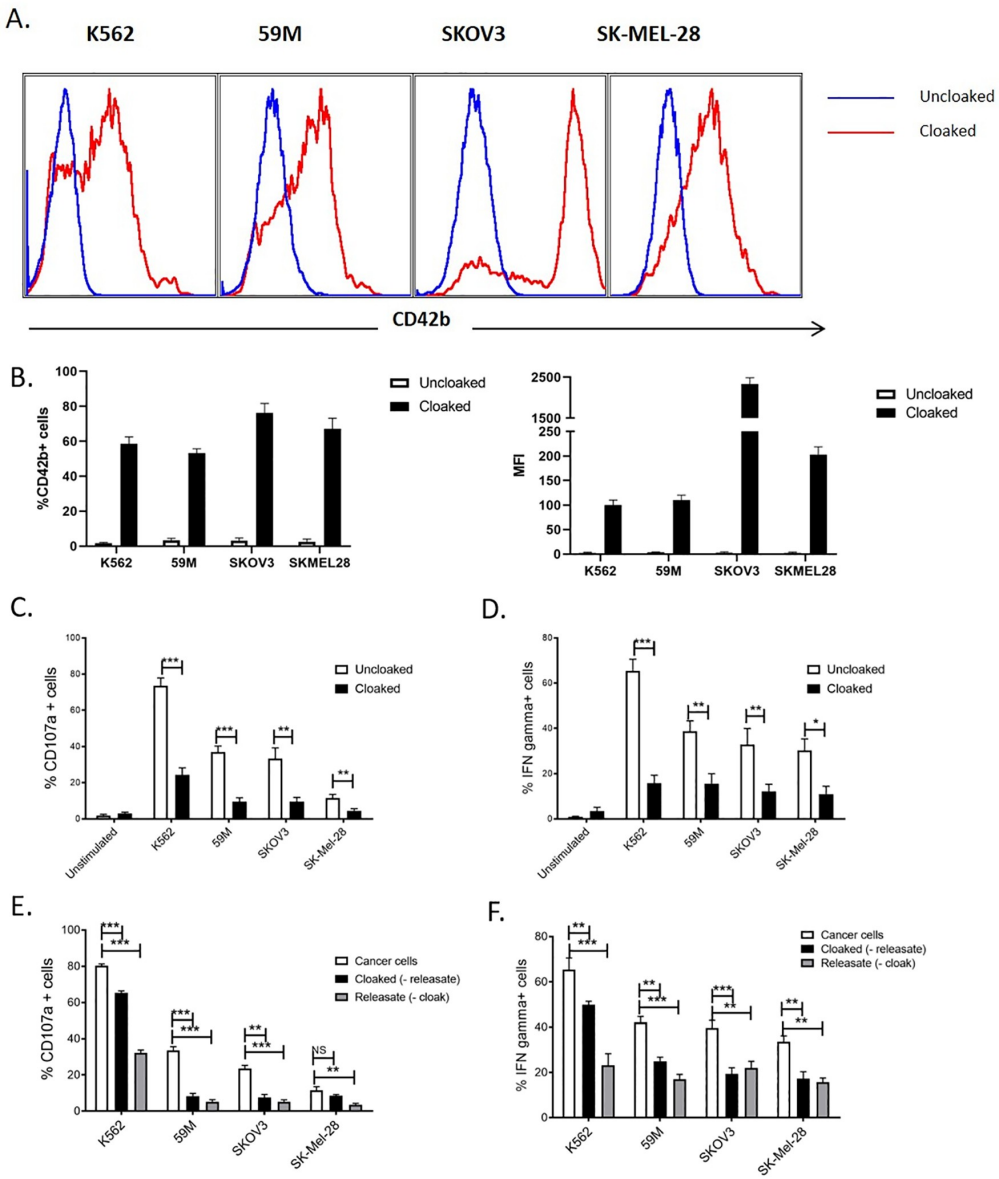
Platelet cloaking inhibits NK cell functions. (A,B) Quantification of platelet cloaking of ovarian and melanoma tumour cells, and the myelogenous leukaemia control cell line K562. Tumour cell lines were co-incubated with and without platelets and analysed for the surface expression of the CD42b platelet specific marker. Expression data are represented by histogram (A), as a percentage of total cells and by mean fluorescent intensity (MFI; B). (C-F) Analysis of the function consequences of platelet cloaking on NK cell functions. NK cell anti-tumour assays were performed by co-incubating PBMCs with tumour cells (cloaked and uncloaked) for 4 hours and measuring CD107a (C,E) and IFNgamma production (D,F) as markers of activation. (E,F) To dissect the respective roles of platelet contact factors (cloaked (minus the releasate)) and soluble factors (releasate (minus the platelet cloak)) platelets and releasate were isolated and used to treat NK cells in NK activation assays as previously described. (C-F) Data analysed by ANOVA—each experiment represents mean±S.E.M. of at least three independent experiments. * = p<0.05, ** = p<0.01, *** = p<0.001.

To establish cloaking of tumour cells, the ovarian cell lines 59M and SKOV3 and the melanoma tumour cells SK-Mel-28 were incubated with platelets and CD42b platelet antigen was measured on tumour cell populations. The K562 cell line, an established target cell of NK cells, was chosen as a positive control. CD42b antigen was detected on tumour cell populations indicating efficient platelet cloaking for each cell line (Fig 1A and 1B; S1 Fig). We subsequently demonstrated robust anti-tumour functions of CD3-CD56+ NK cells (S1 Fig) when challenged with each tumour cell line, quantified by CD107a expression on the NK cell surface and intracellular IFNγ production (Fig 1C and 1D). Ovarian and melanoma tumour cells induced a strong NK interferon gamma production, while melanoma cells poorly induced the CD107a response (Fig 1C and 1D). The presence of the platelet cloak significantly reduced NK cell anti-tumour activity to ovarian and melanoma tumour cells (Fig 1C and 1D). These results suggest that platelet cloaking of tumour cells potently inhibits NK cell effector functions in *in vitro* epithelial cancer models.

### Immune modulation is mediated by both soluble and contact factors

Given that both cell contact factors, such as HLA class-I, and platelet derived soluble molecules, such as TGFβ, are known to alter NK cell effector functions, we addressed the respective roles of soluble and contact factors in ovarian and melanoma tumour cell immune evasion. To study contact factors, PBMCs were incubated with platelet cloaked ovarian and melanoma tumour cells, and our K562 controls, which were washed to remove platelet soluble factors. Washing platelet-cloaked ovarian tumour cells to remove the platelet releasate partially restored NK cell activity and IFNγ production (Fig 1E and 1F). For melanoma tumour cells, CD107a activity was completely restored in the absence of platelet releasate, while IFNγ was unaffected (Fig 1E and 1F). This suggests a role for membrane bound cell-contact factors for ovarian and melanoma tumour cell lines. To examine soluble factors, PBMCs were incubated with tumour cells suspended in platelet releasate (platelet cloaked tumour cell supernatants) in the absence of platelets. Soluble factors potently inhibit NK cell functions for all cell lines. This data demonstrates that both cell contact factors and the platelet releasate induced by both ovarian and melanoma tumour cells is a potent inhibitor of NK cell activity (Fig 1E).

### Platelet cloaking drives an immune decoy mechanism via ADAM proteases

**Fig 2:**
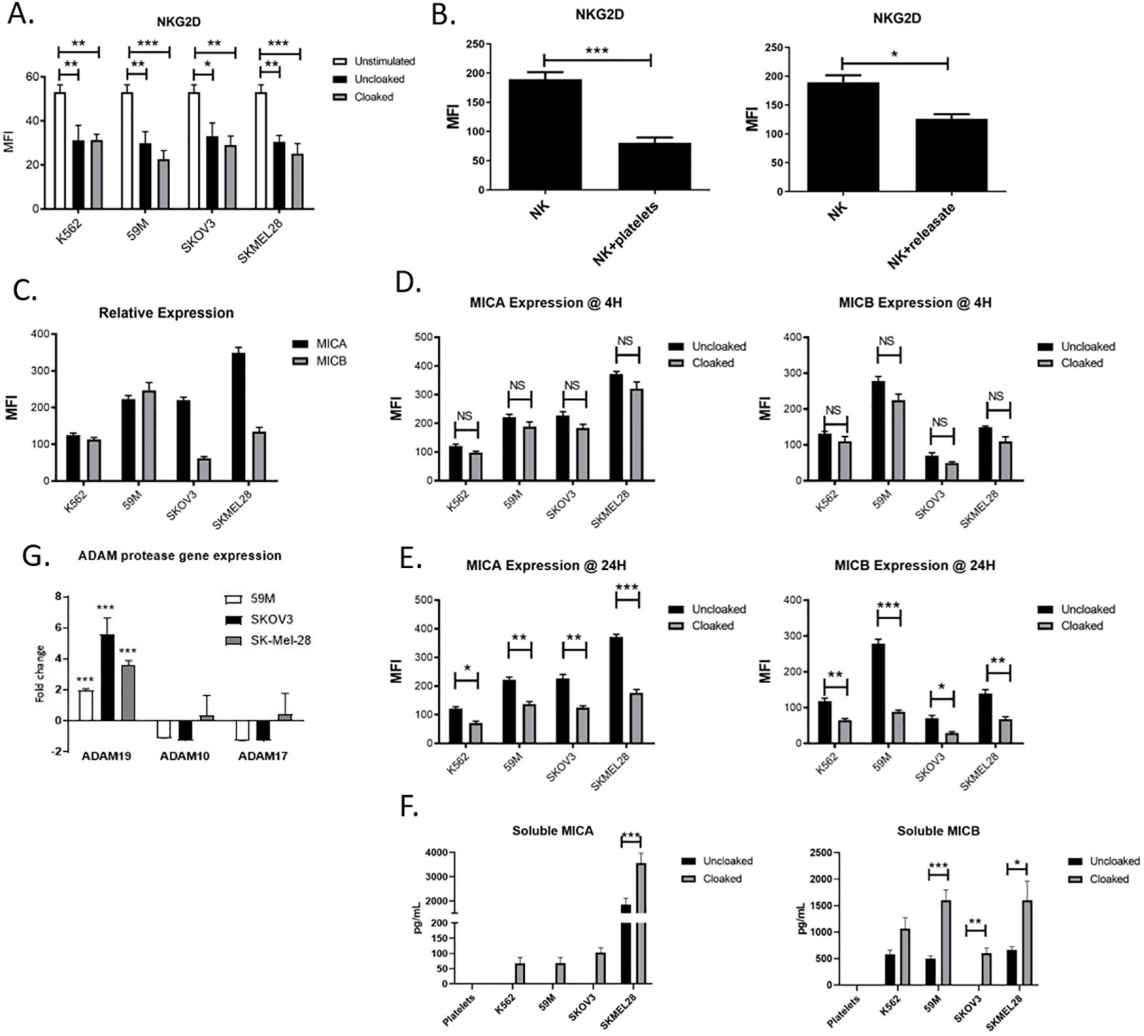
Platelets modulate NKG2D receptor expression by inducing the release of NKG2DL MICA and MICB from the surface of tumour cell lines. (A,B,C) To examine the role of platelet cloaked tumour cells, platelets and TRAP-induced platelet releasate in regulating NKG2D expression, quantification of NKG2D was performed by flow cytometry of PBMCs in the presence or absence of tumour cells, platelet cloaked tumour cells, platelets or platelet releasate for 24 hours. (C) Relative expression of MICA and MICB on tumour cell lines was quantified by MICA/MICB mAbs and flow cytometry (n = 3) (D,E) The role of the platelet cloak on the expression on known NK cell activating ligands MICA and MICB on tumour cells was investigated by measuring baseline expression (clear bars) and comparing this to platelet cloaked tumour cells (filled bars). Tumour cells were incubated for 4 (D) or 24 (E) hours in the presence or absence of platelets and ligand expression was quantified by flow cytometry. (F) Analysis of soluble MICA and MICB in the supernatants of tumour cell lines incubated for 24 hours in the presence or absence of platelets by ELISA. (G) Taqman analysis of platelet cloaked and uncloaked ovarian/melanoma tumour cells for ADAM19, ADAM10 and ADAM17 genes. Values shown are cloaked cells relative to uncloaked cells. (A,B,D) Data analysed by ANOVA—each experiment represents mean±S.E.M. of at least three independent experiments. (G) Data analysed by t-test and represented as standard deviation of at least three independent experiments, * = p<0.05, ** = p<0.01, *** = p<0.001.

The NKG2D-MICA/MICB receptor-ligand system is well established in tumour immune surveillance. We hypothesised that the platelet cloak modulates both receptor and ligand to promote tumour immune evasion. Initially, we confirmed that the axis is functional in NK targeting of our cell lines by NK cell functional assays in the presence/absence of neutralising antibodies to NKG2D (S2 Fig) and the MICA and MICB tumour expressed ligands (S2 Fig). Neutralising each component significantly decreased NK cell anti-tumour activity against each epithelial cancer. We subsequently defined the role of platelet cloaked tumour cells, platelets and platelet releasate (from TRAP activated platelets) in regulating NKG2D expression and NK cell functions. Initial results reveal that both cloaked and uncloaked tumour cells actively suppress NKG2D on the NK cell surface (Fig 2A). Importantly, the decrease in detectable NKG2D on NK cells when co-incubated with tumour cells over 24 hours is well established phenomenon, which we have further demonstrated here. We have therefore dissected the functions of activated platelets and soluble factors in the absence of tumour cells. PBMCs were incubated with either activated platelets (by TRAP) and/or platelet releasate and significantly decreased NKG2D on the surface of NK cells was observed (Fig 2B).

We then examined the effect of the platelet cloak on the tumour cell and its expression of NKG2DL. Each tumour cell line expressed varying levels of MICA and MICB ligand on unstimulated cells (Fig 2C). The expression of surface MICA and MICB was markedly reduced for each epithelial cancer cell line when cloaked with tumour cells (Fig 2D and 2E). The strongest effect was observed for MICA expression on the melanoma cell line and for MICB on the 59M ovarian cells, which expressed the highest basal levels of their respective ligands. To examine the temporal relationship of platelets with decreased expression of tumour ligands, tumour cells were incubated for 4 hours and 24 hours with platelets to examine direct masking of ligand and/or proteolytic cleavage by the platelet (4h) and transcriptional changes in the tumour cell line (24h) (Fig 2D and 2E). Our results suggest that while there is some loss of MICA and MICB from the tumour cell at 4 hours (did not reach statistical significance) the majority of ligand is lost up to 24 hours suggesting that the mechanism is not a rapid steric occlusion but occurs more gradually (Fig 2D and 2E).

A known mechanism of NK cell inhibition is the induced suppression of the NKG2D receptor by soluble NKG2D ligands, specifically MICA and MICB. We incubated NK cells with recombinant MICA and MICB and observed significantly decreased surface NKG2D ligand expression in our system (S3 Fig). It is important to note that the presence of the recombinant molecule in the binding site of the NKG2D receptor may artificially decrease detection with antibody should they compete for binding sites. We therefore characterised the downstream effect of ligand binding to support our hypothesis. NK cells that were pre-treated with recombinant MICA and MICB had decreased anti-tumour functions compared with the IgG-Fc control protein (S3 Fig). This is supported by previous data in which NK cell functions were inhibited when NKG2D receptor functions were neutralised (S2 Fig).

Given that MICA and MICB are lost from the tumour cell surface in a platelet-dependent manner, we hypothesised a role for the platelet in promoting the release of soluble NKG2D ligands into the microenvironment of the cloaked tumour cells. To quantify this, supernatants from tumour cell cultures in the presence or absence of platelets, and platelets alone, were analysed by ELISA for soluble MICA and MICB molecules (Fig 2F). MICA was produced by melanoma cells in the absence of platelets but was not detected for ovarian or control cells. (Fig 1C). Platelet cloaking induced the release of MICA from all cell lines, however this only reached significance for our melanoma cells. Similarly, the presence of the platelet cloak induced secretion of soluble MICB from all cell lines, most notably from the 59M and SKOV-3 ovarian cell lines, demonstrating a platelet-dependent immune evasion mechanism.

The temporal relationship of cloaking with sNKG2DL release indicates tumour cell gene regulation as mechanistically important. Taqman relative PCR of cloaked and uncloaked tumour ovarian cells revealed that ADAM19 is upregulated in platelet cloaked ovarian and melanoma cells compared with uncloaked cells (Fig 2G). Interestingly, ADAM10 and ADAM17 are not upregulated in the tumour cell, suggesting a novel and distinct process to regulate NKG2DL cleavage on the tumour cell surface. Our results demonstrate that platelets specifically upregulate genes of a protease family known to cleave NKG2DL ligands and suggests a potential role for tumour derived ADAM proteases in NKG2DL cleavage.

**Fig 3:**
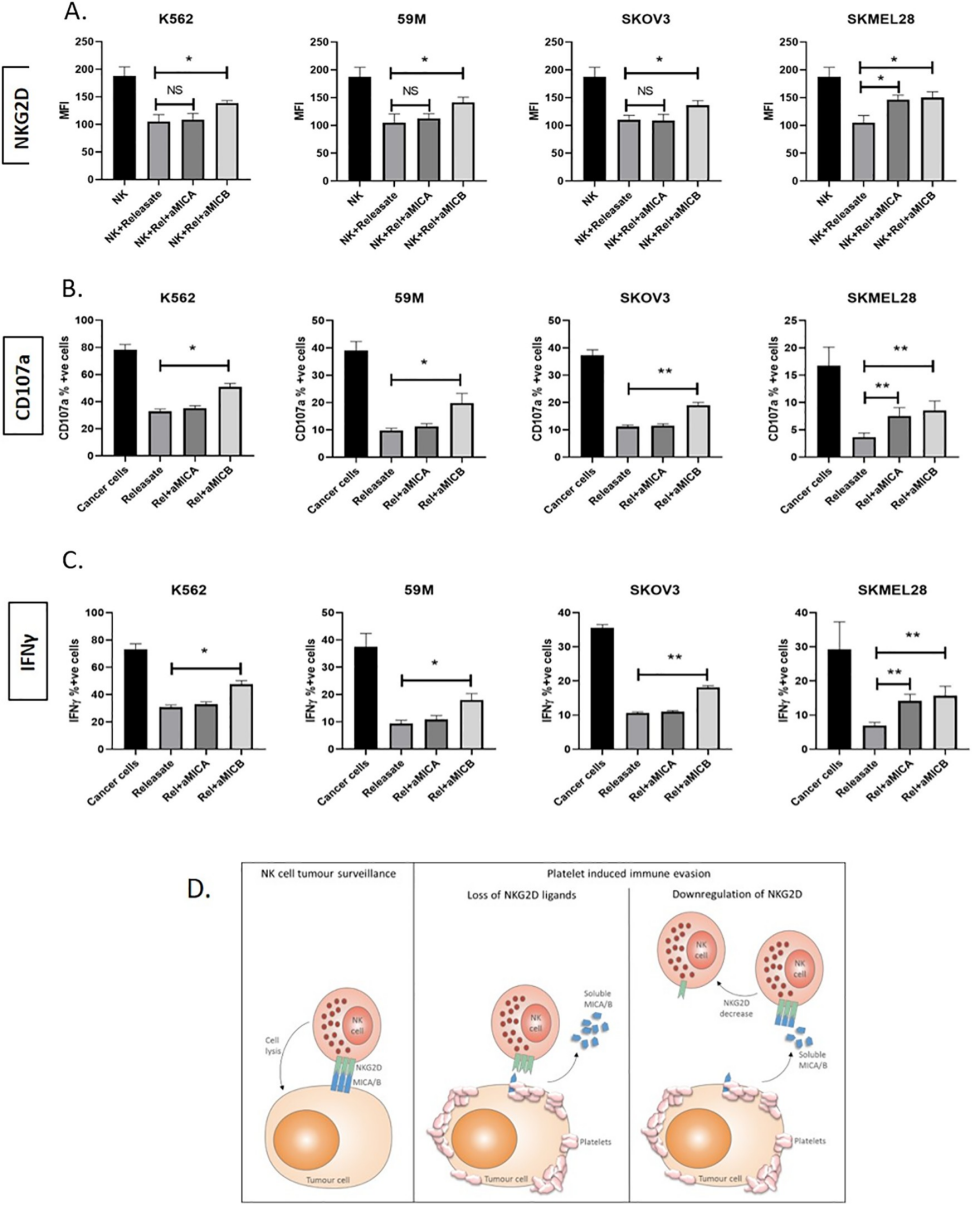
MICA and MICB in the releasate modulate NKG2D expression and NK cell functions. (A) To examine the role of MICA and MICB from the platelet releasate (from platelet cloaked tumour cells) in regulating NKG2D and NK cell functions, the platelet releasate was neutralised using monoclonal antibodies against MICA and MICB as previously described. NKG2D expression (A) and NK cell functions (CD107a (B) and IFNgamma (C)) were quantified following standard 4h NK cell function assays by flow cytometry and results are expressed as the mean fluorescent intensity (MFI; A) or as a percentage of unstimulated or IgG control (B and C). (D) Schematic representation of the platelet sNKG2DL mediated decoy mechanism of tumour immune evasion (A-C) Data analysed by ANOVA—each experiment represents mean±S.E.M. of at least three independent experiments. * = p<0.05, ** = p<0.01, *** = p<0.001

By treating releasate from platelet cloaked tumour cells that contained platelet soluble factors plus tumour cell soluble factors, with neutralising antibodies to MICA and MICB the suppression of NKG2D (Fig 3A), degranulation (Fig 3B) and pro-inflammatory cytokine (Fig 3C) production was partially rescued. Interestingly, rescue with anti-MICA antibody was only achieved for the melanoma cells, consistent with our finding that MICA is released only from melanoma cells in significant quantities. Additionally, rescue was observed for all cell types when MICB was neutralised as MICB was present in the releasate from each cell line.

Together these results reveal a decoy mechanism to promote tumour cell evasion from NK cell surveillance (Fig 3D).

### Platelet cloaking disrupts the CD226/CD96-CD112/CD155 tumour recognition axis

Like NKG2D, CD226 and CD96 are receptors expressed on the surface of NK cells known to be important for tumour immune surveillance. The ligands for the CD226 and CD96 receptors on the tumour cell are CD112 and CD155. We confirmed and quantified their expression on the ovarian and melanoma cell lines by immunofluorescence using anti-CD112 and anti-CD155 specific monoclonal antibody based flow cytometry and each of our ovarian and melanoma tumour cell lines express both CD112 and CD155 (S4 Fig).

**Fig 4:**
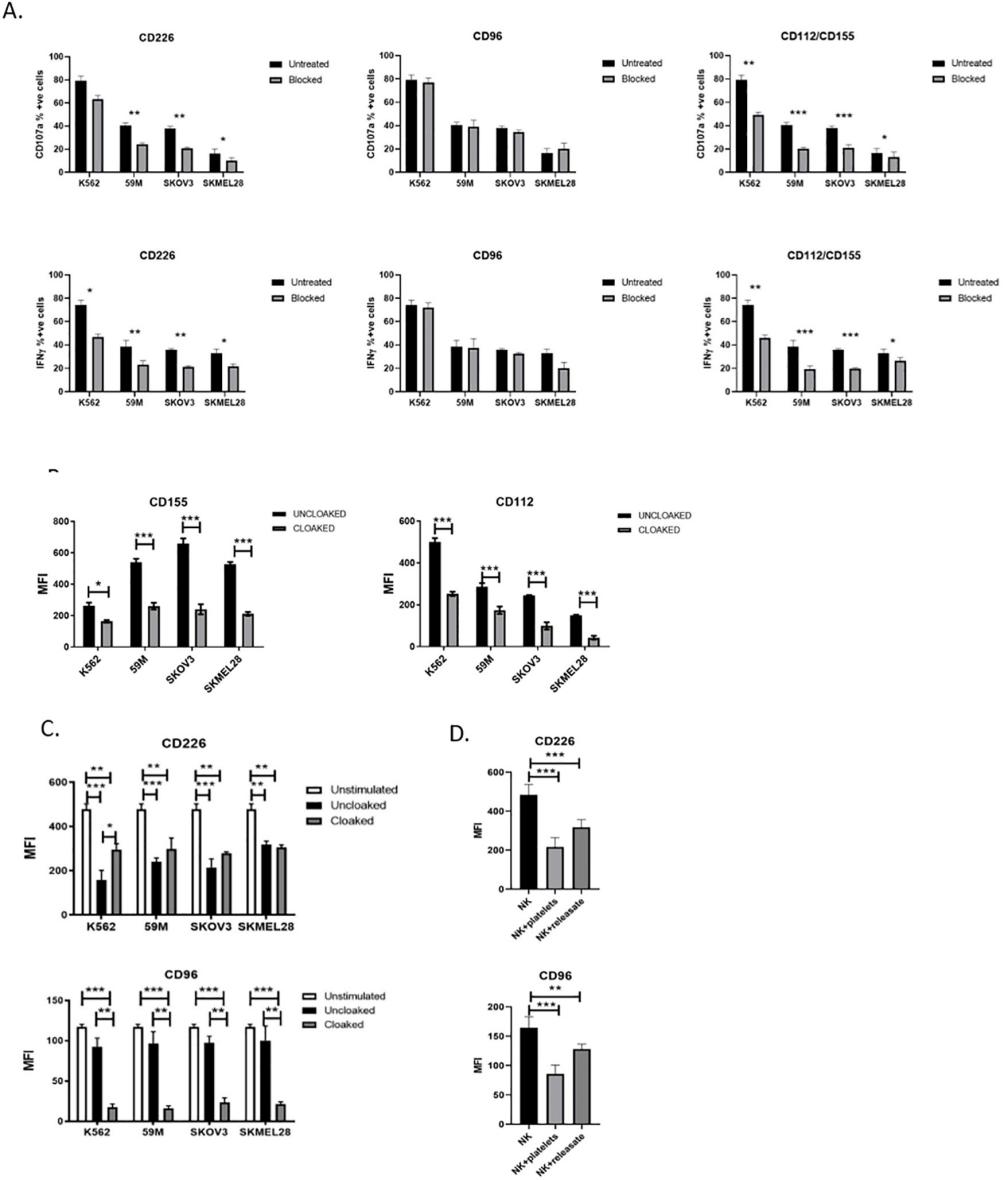
Platelets disrupt the CD226/CD96-CD112/CD155 NK axis for NK cell targeting of tumour cell lines. (A) Blocking assays using mAb against CD226, CD96 or CD155/CD112 in standard anti-tumour assays (CD107a surface expression and IFNy production) to analyse the role of each molecule in NK cell targeting of tumour cell lines. (B) The role of the platelet cloak on the expression on known NK cell activating ligands CD112 and CD155 on tumour cells was investigated by measuring baseline expression (clear bars) and comparing this to platelet cloaked tumour cells (filled bars). Tumour cells were incubated for 24 hours in the presence or absence of platelets and ligand expression was quantified by flow cytometry. (C) Analysis of the effect of tumour cells with and without the platelet cloak on expression of the NK cell receptors CD226 and CD96. PBMCs (clear bars) were co-incubated with uncloaked (filled bar) and cloaked (grey bars) tumour cells for 24 hours and expression of CD226 and CD96 were quantified by flow cytometry. (D) To dissect the role of soluble factors, experiments were repeated as above with TRAP-activated degranulating platelets (+platelets group) and releasate from TRAP-activated degranulated platelets that was cleared of platelet cellular material (+releasate group). (A-D) Data analysed by ANOVA—each experiment represents mean±S.E.M. of at least three independent experiments. * = p<0.05, ** = p<0.01, *** = p<0.001.

To investigate if the CD226/CD96-CD155/CD112 tumour cell recognition system is functional in the context of our ovarian and melanoma tumour cells we blocked each component with commercial mAb and performed NK cell functional assays (Fig 4 and S4 Fig). Blocking CD226 on the NK cell induced a marked inhibition of NK functions for both activity and cytokine production, with strongest effects seen for the epithelial cancer cell lines (Fig 4A). Neutralising CD96 on the NK cell (Fig 4A), or the ligands CD155 or CD112 (S4 Fig) on the tumour cell, did not disrupt NK cell targeted killing of our tumour cell lines. However, dual blocking of CD155 and CD112 ligands demonstrated marked inhibition of NK anti-tumour functions (Fig 4A). This highlights complexity and redundancy within the system.

Given the role of platelets in regulating tumour cell expressed ligands and NK cell expressed receptors in the NKG2D-MICA/MICB system, we hypothesised that the CD226/CD96-CD112/CD155 axis would be regulated in a similar manner. We observed that tumour cell lines cloaked with platelets had a significant decrease in expression of CD112 and CD155 (Fig 4B). As previously shown, platelet cloaked tumour cells are less immunogenic than uncloaked cells and by blocking access to tumour cell ligands (using monoclonal antibodies) NK cell degranulation and cytokine synthesis are inhibited (Fig 4A and S4 Fig). This immune evasion mechanism is mediated by platelets and actively disrupts the CD226/CD96-CD112/CD155 axis.

We further confirmed that the platelet cloak downregulates NK cell CD226 and CD96 (Fig 4C). CD226 was inhibited when incubated with uncloaked tumour cells alone, a phenomenon that has been published previously (Fig 4C). There is a paradoxical increase in CD226 with platelet cloaked tumour cells, an artefact from CD226+ activated platelets sticking to NK cells (Fig 4C). Interestingly, NK activity was unaffected at 24 hours compared with 4 hours incubation suggesting that decreased CD226 in not mechanistic in inhibiting NK cells in this scenario. To dissect further the role of the activated platelet and releasate, PBMCs were co-incubated with either TRAP activated platelets or platelet releasate and NK receptor expression quantified by flow cytometry (Fig 4D). CD226 expression on the cell surface was decreased by both activated platelets and platelet releasate (Fig 4D). More potent inhibition with platelet material suggests that molecules from the platelet surface have regulatory functions while soluble molecules are also active as demonstrated by a potent suppression of CD226 by the releasate (Fig 4D). A similar effect was observed for CD96 with significantly decreased expression in response to platelet cloaked tumour cells, platelets and releasate suggesting a role for both in CD96 suppression, suggesting that CD226 and its CD96 co-receptor are suppressed in concert (Fig 4C and 4D). The regulation of CD96 on the NK cell is, unlike CD226, independent of the suppressive effects of the tumour cell alone.

### TGFβ from platelets inhibits CD226 but not CD96 on NK cells

**Fig 5:**
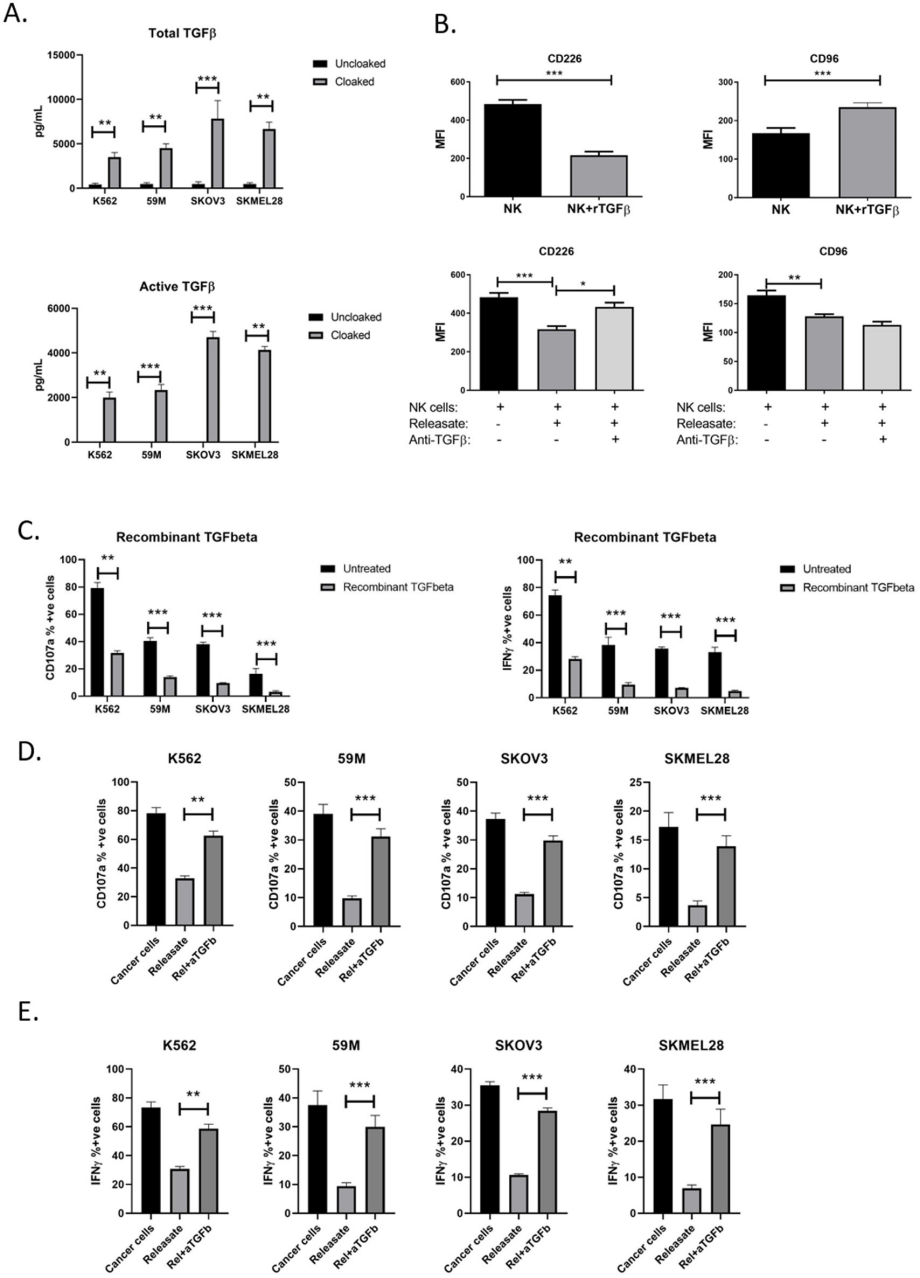
TGFbeta is an active soluble molecule in platelet derive immune evasion of circulating tumour cells. (A) Quantification of TGFbeta (total and active) released from cloaked tumour cells (filled box) compared with uncloaked cells (clear box) using ELISA duo-set analysis of supernatants of tumours cells incubated for 4 hours in the presence or absence of platelets. (B) To dissect the role of TGFbeta, recombinant TGFbeta1 molecule was used to pre-treat NK cells for 24 hours and CD226 and CD96 receptor expression was quantified by flow cytometry. To confirm the in vitro role of platelet derived TGFbeta, neutralising antibody against TGFbeta was used to pre-clear supernatants and receptor expression was compared with untreated cells. (C) Functional assays to confirm the effect of TGFbeta on NK cell anti-tumour functions were performed by pre-treating NK cells with recombinant TGFbeta and measuring CD107a surface expression and IFNgamma production as NK cell activation markers. (D,E) Analysis of the role of TGFbeta in platelet releasate on CD107a expression (D) and IFNgamma production (E) by comparison of the effect of TRAP-induced platelet releasate versus releasate that has been cleared of TGFbeta by neutralising antibody. (C-E) Results represent the detection of the activation markers as a percentage of the average response of untreated NK cells to each individual tumour cell line. (A-E) Data analysed by ANOVA—each experiment represents mean±S.E.M. of at least three independent experiments. * = p<0.05, ** = p<0.01, *** = p<0.001.

TGFβ has established functions in NKG2D receptor down-regulation and NK cell anti-tumour functions. TGFβ is released from activated platelets as demonstrated by ELISA assays performed on tumour cells in the presence or absence of platelets (Fig 5A). Recombinant TGFβ downregulated CD226 expression on NK cells. Interestingly, there was a significant increase in CD96 expression (Fig 5B). Additionally, by neutralising TGFβ in the platelet releasate (from platelet cloaked tumour cells) using anti-TGFβ antibody we observed rescue of CD226 suppression, supporting TGFβ dependant regulation of this receptor. No significant effect was observed for CD96 indicating that this receptor is regulated independently of TGFβ. By treating NK cells with recombinant TGFβ their functions could be suppressed (Fig 5C), which was supported further by neutralising TGFβ in the releasate to rescue NK cell suppression (Fig 5D and 5E). Together these data demonstrate that TGFβ suppresses NK cell functions via downregulation of CD226 on the NK cell surface.

## Discussion

In the bloodstream, cancer cells (also known as circulating tumour cells or CTCs) encounter many challenges that are overcome, in part, by the formation of CTC-platelet clusters mediated by coagulation factors released both by the tumour cell and the activated platelets [14]. CTCs must evade immune clearance by NK cells in peripheral circulation. NK cells express receptors that efficiently bind tumour cell antigens and release cytotoxic granules to neutralise tumour cells in the blood. However, as previously reported, CTCs induce the release of immune-suppressive molecules from platelets to inhibit NK cells and evade detection [7, 15]. Furthermore, platelets have been shown to transfer their major histocompatibility complex class I to tumour cells, thereby conferring ‘self’ status and contributing to immune evasion [6]. The interactions between tumour cells and platelets are complex, particularly the role of platelets in facilitating immune evasion of CTCs, similar to that previously identified for germ cell, prostate and colon cell lines [7].

In this study, we examined the effects of platelets and their releasate on two different models of metastatic disease; using low trafficking ovarian cancer cells that spread primarily through the abdominal cavity in ascitic fluid and less commonly through peripheral vasculature, and high trafficking malignant melanoma cells that spread predominantly via the lymphatic and vascular systems. We predicted that cells with such disparate metastatic strategies would vary in their basic molecular anti-immune functions. Using flow cytometry, we confirmed the capacity of ovarian and melanoma tumour cells to efficiently activate and adopt a cloak of platelets and induce the release of modulatory molecules, including TGFβ. We have subsequently shown that the molecules contained within the platelet releasate are potent inhibitors of NK cells. We also demonstrated the conserved roles of contact factors from cell-cell interaction and soluble factors from the platelet releasate in suppressing NK cell anti-tumour functions to tumour cells that spread haematogenously (high trafficking) and transcoelomically (low trafficking).

The interaction between NKG2D on the NK cell and NKG2DL (MICA and MICB) on the tumour cell activates NK cell anti-tumour functions. As NKG2DL are expressed only on stressed cells, including malignantly transformed cells, this axis represents a targeted mechanism for immune clearance of cancer cells. Interestingly, prolonged exposure of NK cells to ligand expressing tumour cells results in decreased levels of NKG2D on the NK cell. This was an important consideration in our study and we therefore examined the functions of platelets and the soluble factors in isolation from tumour cells. In our study, platelet releasate from cloaked ovarian and melanoma tumour cells suppressed NKG2D on NK cells, a phenomenon previously reported for prostate and colon cancer cells [7]. Platelets acted on the tumour cell to decrease detectable surface NKG2DL, altering the ‘stressed or non-self’ to ‘self’ phenotype. Platelet cloaking subsequently reduced the immunogenicity of tumour cells to NK cells, promoting immune evasion. We supported this finding by demonstrating decreased NK cell recognition and reactivity to tumour cells that were neutralised with antibodies against the NKG2DL, MICA and/or MICB.

TGFβ from the platelet is a well-established mechanism of suppression of NKG2D receptor surface expression and NK cell function. However, we observed that NKG2D, and thus NKG2D function, is more potently suppressed by the platelet releasate than by recombinant TGFβ (S2 Fig). We, therefore, hypothesised that solubilised NKG2DL contributes to NKG2D suppression. In our study, ovarian and melanoma tumour cells with a platelet cloak could be induced to release soluble NKG2DL into the tumour cell microenvironment and that NKG2D was actively suppressed using recombinant MICA and MICB proteins. A recent study demonstrates the impact of platelet cloaking on the shedding of NKG2D ligands, which supports these findings [16]. Additionally, by neutralising soluble MICA and MICB in the platelet releasate we partially restored NKG2D suppression and function. Neutralising MICA was effective only for melanoma cells, which release soluble MICA in high doses in a platelet-independent manner. This perhaps suggests that the high trafficking melanoma cells release high concentrations of soluble MICA to evade NK cell detection and attack, providing an explanation for the lesser capacity of NK cells to mount an immune response to this cell line. Soluble MICA for ovarian cancer cells, while induced by platelet cloaking, saw only modest increases in levels in the microenvironment and is therefore unsurprising that neutralising sMICA in the releasate has no measurable effect. Conversely, however, neutralising MICB in the releasate restored NKG2D expression for each cell line which correlates with the platelet induced release of soluble MICB from tumour cells, previously described. This suggests a conserved role for MICB in high and low-trafficking cells, while MICA appears functional only in the high-trafficking cell type.

A role for the ADAM proteases in the shedding of MICA and MICB has been shown previously [16, 17]. To examine the role of platelet-surface ADAM proteases we defined the temporal relationship of platelet cloaking with shedding of MICA and MICB from the surface. We found that while low levels of shedding are detectable at shorted time intervals (attributed to direct cleavage by platelet-surface ADAM10 and ADAM17), a much stronger effect was observed at 24 hours incubation. We subsequently examined ADAM10, ADAM17 and ADAM19 expression in the tumour cell and found that while ADAM10/17 are not induced by platelet cloaking, ADAM19 is potently induced. We suggest that ADAM19 plays an important functional role in MICA and MICB cleavage and we aim to address this further in future studies. Indeed, the detection of soluble MICA and MICB in the serum of patients with advanced hepatocellular carcinoma [18] and oral squamous cell carcinoma [19] respectively represents a marker of poorer prognosis, while increased soluble MICA and decreased NKG2D levels are poor prognostic markers in pancreatic cancer [20]. Our results demonstrate an immune decoy mechanism for tumour cell escape from Natural Killer cells in the peripheral circulation and identify a novel mechanistic target in the metastatic cascade for cancer immunotherapy.

NK cell immunoglobulin receptors CD226 and CD96 that interact with the nectin-like ligand CD112 and the polio-virus receptor CD155 on target cells are emerging as important mediators of NK cell anti-cancer functions [21–23]. The function of CD226 in recognition of tumour cells by NK cells has been previously examined [24]. In our study, we initially demonstrated that CD226 was functional in NK targeting of our melanoma and ovarian tumour cell lines. This was achieved by observing the negative functional impact of blocking the CD226 receptor with commercial mAb. Interestingly, neutralising CD96, a known adhesion molecule had no significant effect on NK cell functions. This is supported by previously published data [24]. CD96 is less well understood. Its primary ligand is CD155 and recent studies of murine CD96 have shown it to be a competitive inhibitor of CD226, competing for binding to CD155 with a stronger binding affinity for the ligand and directly inhibiting NK cell degranulation and cytokine production as a result [21]. However, CD96 acts as an adhesion molecule facilitating NK activity in the human context, and is therefore described as an NK cell activating receptor [25]. Evolutionarily divergent functions of the murine and human CD96 molecule has been considered by experts as an explanation for the differing reports and more work is required to fully define CD96 in the human context. The functional complexities of CD96 in tumour immune surveillance are intriguing, however, they are beyond the scope of this study and we intend to focus on these in further studies. Supporting a role for CD226 and CD96 in the context of tumour evasion, a study of patients with pancreatic cancer demonstrated that CD226+CD96+ NK cells are deficient in patients versus healthy controls, while TIGIT was not altered [26]. Additionally, CD226 and CD96 upregulation was observed on NK cells of relapse free breast cancer patients and CD226 was found to be decreased on anergic NK cells associated with lung cancer [27–29].

Given that the axis is active in immune surveillance of both melanoma and ovarian tumour cells, we established that platelet cloaked tumour cells, activated platelets and platelet releasate all function to suppress expression of CD226 and CD96 on the NK cell surface. Furthermore, we observed that platelet cloaked tumour cells have significantly decreased expression of the CD112 and CD155 ligands that activate CD226/CD96. This mimics the dual functionality of the platelet in the NKG2D/NKG2DL system to inhibit both immune receptors and the tumour cell marker. The role of TGFβ in modulating NK cell receptor function is established for NKG2D [7], and herein we have described a similar role for CD226. Recombinant TGFβ suppressed CD226 expression and NK cell functions and this was supported by neutralising TGFβ in the platelet releasate to partially rescue suppression. TGFβ significantly increased CD96 expression on NK cells, suggesting that CD96 is regulated independently of TGFβ. This novel role for TGFβ may aid in the development of successful anti-TGFβ or anti-platelet strategies in battling metastatic disease.

Taken together, these findings indicate a profound and systematic inhibition of innate immune surveillance by platelet cloaking or elaboration of platelet releasate from cancer-primed platelets and provide potential future chemotherapeutic targets for metastatic disease.

## Supporting information

S1 Fig Representative gating strategy. (A) Gating of tumour cell populations from free platelets. FSC/SCC plots of SKOV3 cells, platelets and cloaked SKOV3 cells demonstrates two distinct populations. (B) Tumour cell gate (P11) stained with CD42b APC antibody. Isotype control, SKOV3, platelet and cloaked SKOV3 samples demonstrate the low free platelet contamination in the tumour cell population and the positive and negative populations for cloaked tumour cells. (C) A lymphocyte gate was applied to whole PBMCs from healthy donors. Lymphocytes were gated for NK cells (CD3-CD56+ cells). (D) CD3-CD56+ NK cells were treated with cancer cells and cloaked cancer cells (cancer cells + platelets) and analysed for CD107a expression.
(TIF)

S2 Fig Neutralising the NKG2D-NKG2DL axis inhibits NK cell functions. (A) Quantifying the capacity of monoclonal antibodies to neutralise NKG2D receptor on NK cells and MICA and MICB ligands on tumour cell lines. PBMCs and tumour cell lines were incubated for 1 hour at room temperature in the presence or absence of 1μg/mL of respective mAb and subsequently stained with fluorescent antibodies to quantify molecular blockade compared with untreated cells. For tumour cell lines, the clear box represents staining in the absence of mAb blockade and the filled box represents neutralised cells. (B) Given the satisfactory neutralisation of surface molecules, the cells were used in standard anti-tumour assays (CD107a surface expression and IFNgamma production) to analyse the role of each molecule (and indeed, a combination of molecules) in NK cell targeting of tumour cell lines. (C) Expression of NKG2D on NK cells. NKG2D was potently suppressed but both platelet releasate and TGFbeta recombinant protein, with significant inhibition with releasate compared with recombinant protein. (A,B,C) Each experiment represents mean±S.E.M. of at least three independent experiments. * = p<0.05, ** = p<0.01, *** = p<0.001.
(TIF)

S3 Fig The role of soluble MICA and MICB in NKG2D expression and NK cell functions. (A) Expression of NKG2D on NK cells post-treatment with recombinant MICA or MICB for 24 hours. (B and C) NK cells were also functionally analysed for CD107a expression and IFNy production. Results are expressed as a percentage of control in the presence of IgG control for each cell line. (A-C) Data analysed by ANOVA—each experiment represents mean±S.E.M. of at least three independent experiments. * = p<0.05, ** = p<0.01, *** = p<0.001.
(TIF)

S4 Fig Quantifying expression and function of CD112 and CD155 ligands on tumour cell lines. (A) Quantifying CD112 and CD155 ligands on tumour cell lines using fluorescent mAb and flow cytometry (B) Monoclonal antibodies against CD155 or CD112 were used to block NK cell targeting of tumour cell lines. NK cells were co-incubated with tumour cells in the presence or absence of tumour cells that were pre-treated with neutralising antibodies and degranulation and cytokine production was quantified. Results are expressed as a percentage increase or decrease of neutralised conditions compared with untreated cells. (C) 24 hour timepoint for NK reactivity. CD107a and IFN gamma quantification of NK cells that were incubated for 24 hours with either tumour cells alone or with cloaked tumour cells (A,B,C) Data analysed by ANOVA—each experiment represents mean±S.E.M. of at least three independent experiments. * = p<0.05, ** = p<0.01, *** = p<0.001.
(TIF)
