# Supplementary figures and images for: Suppression of Natural Killer cell NKG2D and CD226 anti-tumour cascades by platelet cloaked cancer cells: Implications for the metastatic cascade
Source: PLoS One. 2019 Mar 25;14(3):e0211538. doi: 10.1371/journal.pone.0211538 (PMC6433214; doi:10.1371/journal.pone.0211538)

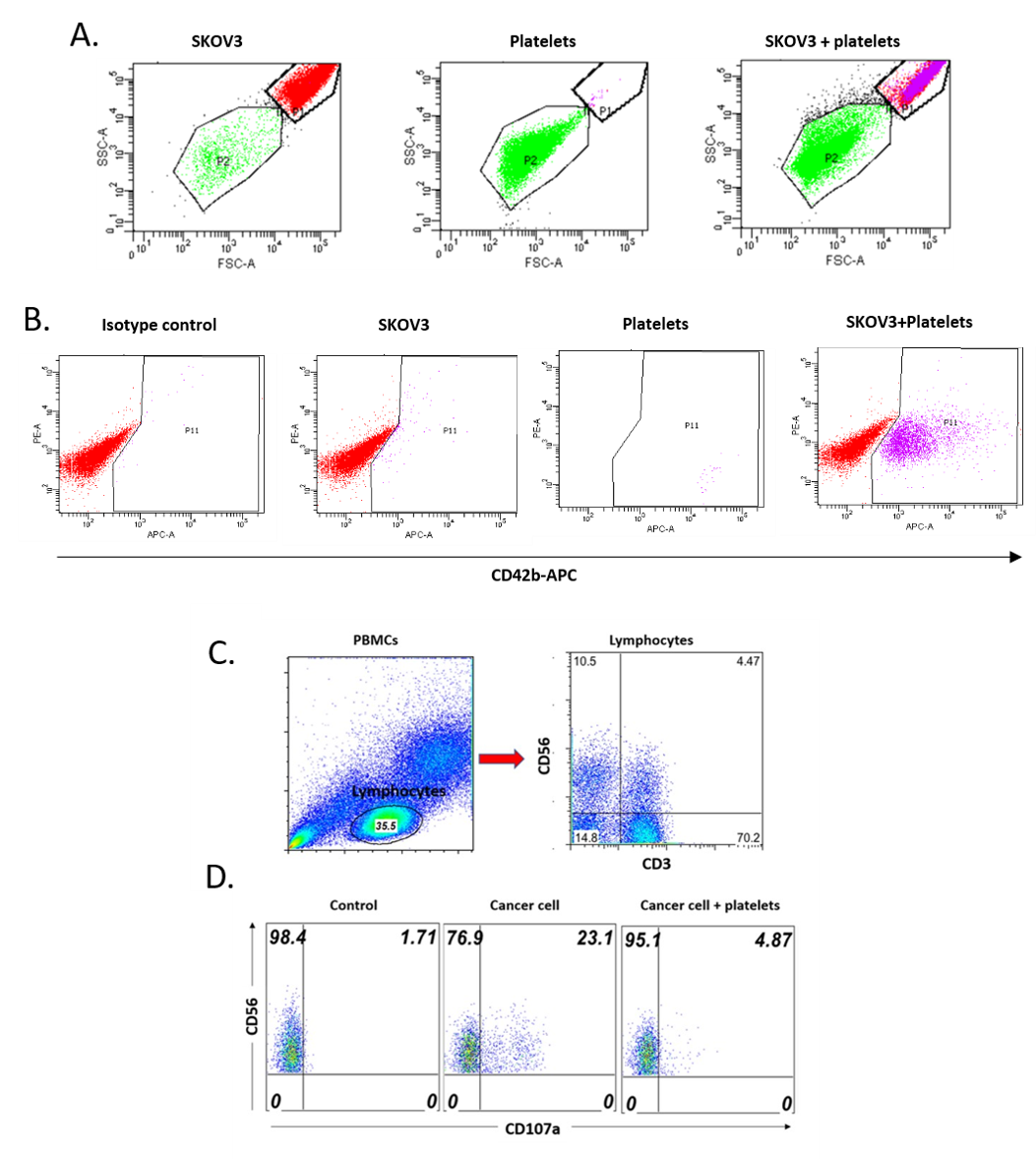

Supplement: S1 Fig — (A) Gating of tumour cell populations from free platelets. FSC/SCC plots of SKOV3 cells, platelets and cloaked SKOV3 cells demonstrates two distinct populations. (B) Tumour cell gate (P11) stained with CD42b APC antibody. Isotype control, SKOV3, platelet and cloaked SKOV3 samples demonstrate the low free platelet contamination in the tumour cell population and the positive and negative populations for cloaked tumour cells. (C) A lymphocyte gate was applied to whole PBMCs from healthy donors. Lymphocytes were gated for NK cells (CD3-CD56+ cells). (D) CD3-CD56+ NK cells were treated with cancer cells and cloaked cancer cells (cancer cells + platelets) and analysed for CD107a expression. (TIF) [file pone.0211538.s001.tif]

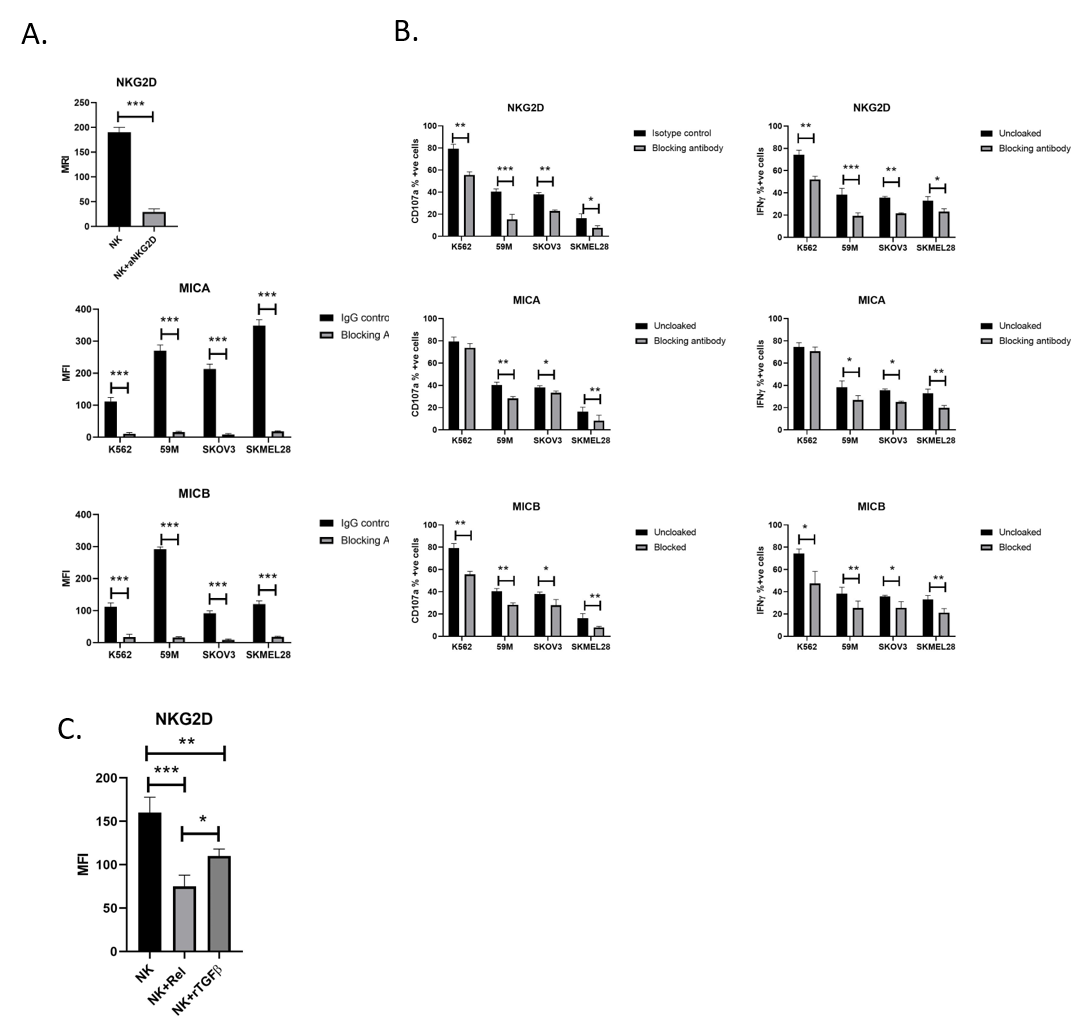

Supplement: S2 Fig — (A) Quantifying the capacity of monoclonal antibodies to neutralise NKG2D receptor on NK cells and MICA and MICB ligands on tumour cell lines. PBMCs and tumour cell lines were incubated for 1 hour at room temperature in the presence or absence of 1μg/mL of respective mAb and subsequently stained with fluorescent antibodies to quantify molecular blockade compared with untreated cells. For tumour cell lines, the clear box represents staining in the absence of mAb blockade and the filled box represents neutralised cells. (B) Given the satisfactory neutralisation of surface molecules, the cells were used in standard anti-tumour assays (CD107a surface expression and IFNgamma production) to analyse the role of each molecule (and indeed, a combination of molecules) in NK cell targeting of tumour cell lines. (C) Expression of NKG2D on NK cells. NKG2D was potently suppressed but both platelet releasate and TGFbeta recombinant protein, with significant inhibition with releasate compared with recombinant protein. (A,B,C) Each experiment represents mean±S.E.M. of at least three independent experiments. * = p<0.05, ** = p<0.01, *** = p<0.001. (TIF) [file pone.0211538.s002.tif]

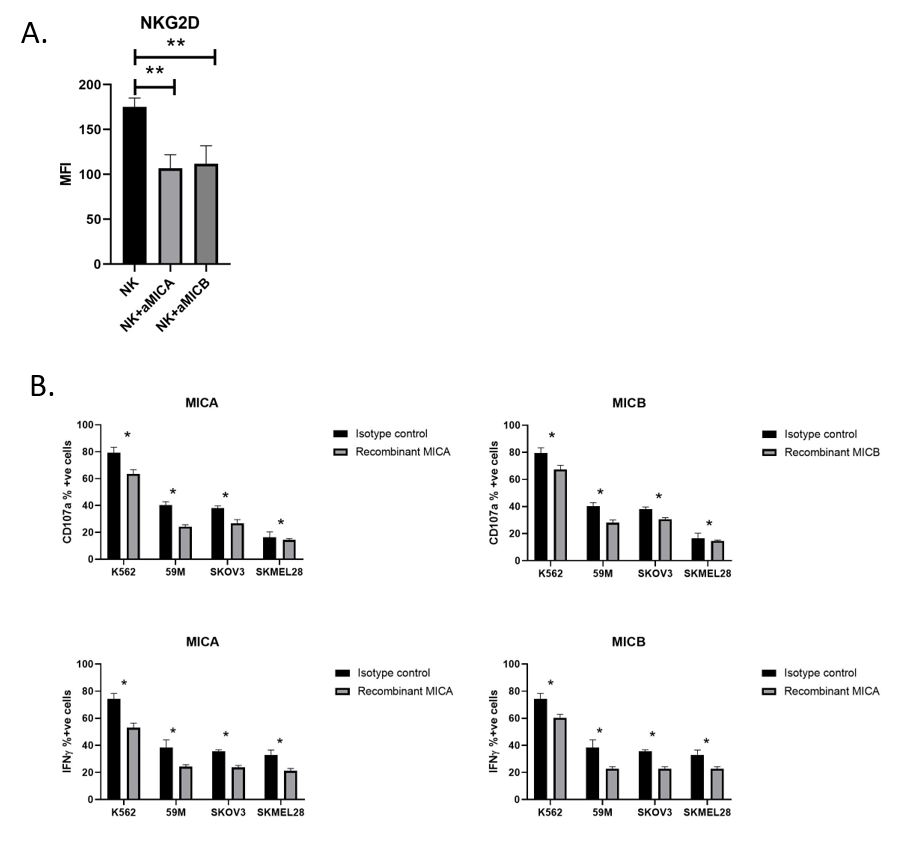

Supplement: S3 Fig — (A) Expression of NKG2D on NK cells post-treatment with recombinant MICA or MICB for 24 hours. (B and C) NK cells were also functionally analysed for CD107a expression and IFNy production. Results are expressed as a percentage of control in the presence of IgG control for each cell line. (A-C) Data analysed by ANOVA—each experiment represents mean±S.E.M. of at least three independent experiments. * = p<0.05, ** = p<0.01, *** = p<0.001. (TIF) [file pone.0211538.s003.tif]

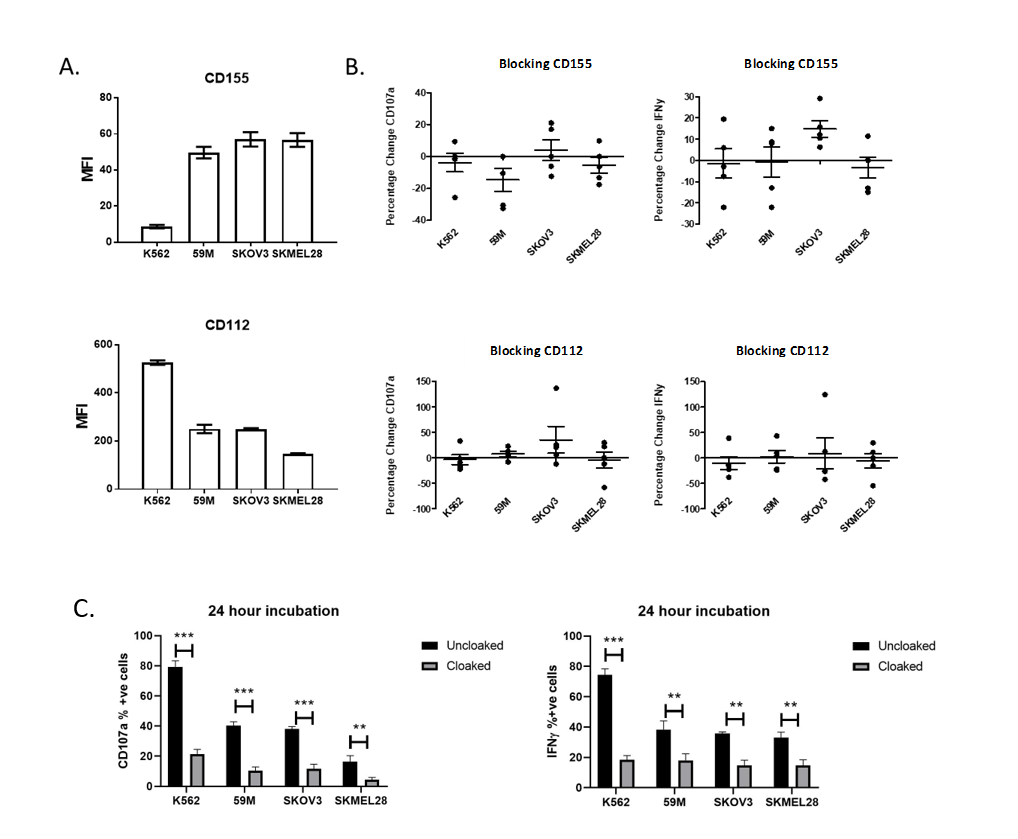

Supplement: S4 Fig — (A) Quantifying CD112 and CD155 ligands on tumour cell lines using fluorescent mAb and flow cytometry (B) Monoclonal antibodies against CD155 or CD112 were used to block NK cell targeting of tumour cell lines. NK cells were co-incubated with tumour cells in the presence or absence of tumour cells that were pre-treated with neutralising antibodies and degranulation and cytokine production was quantified. Results are expressed as a percentage increase or decrease of neutralised conditions compared with untreated cells. (C) 24 hour timepoint for NK reactivity. CD107a and IFN gamma quantification of NK cells that were incubated for 24 hours with either tumour cells alone or with cloaked tumour cells (A,B,C) Data analysed by ANOVA—each experiment represents mean±S.E.M. of at least three independent experiments. * = p<0.05, ** = p<0.01, *** = p<0.001. (TIF) [file pone.0211538.s004.tif]
